# Cocaine Induced Pleural and Pericardial Effusion Syndrome

**DOI:** 10.1155/2015/321539

**Published:** 2015-03-30

**Authors:** Shehabaldin Alqalyoobi, Omkar Vaidya, Al-Ma'Mon Abu Ghanimah, Ahmed Elkhanany, Ashraf Gohar

**Affiliations:** Internal Medicine Department, University of Missouri-Kansas City School of Medicine, Kansas City, MO 64108, USA

## Abstract

A 42-year-old African American female with chronic cocaine use for 20 years, presented with two-day history of exertional shortness of breath and pleuritic chest pain. She was admitted three years back with acute kidney injury and skin rashes. At that time, skin biopsy was consistent with leukocytoclastic vasculitis and renal biopsy revealed proliferative glomerulonephritis. She responded to oral prednisone and mycophenolate with complete recovery of her kidney functions. Skin rash was waxing and waning over the last two years. On the second admission, patient was found to have large pleural effusion on computerized tomography scan and pericardial effusion on echocardiogram as shown in the figures. Pleural fluid analysis was exudative. Her serology was negative for ANA (antineutrophilic antibody) and anti-dsDNA (double stranded DNA). Complements levels were normal. She had positive low titers of ANCA levels. The patient was started on a course of prednisone for 6 months. Her pleural and pericardial effusion resolved completely on follow-up imaging with computerized tomography scan and echocardiogram. This case is unique since the pericardial and pleural effusions developed without any other etiology in the setting of cocaine; hence, we describe this clinical syndrome as cocaine induced pleural and pericardial effusions syndrome (CIPP).

## 1. Case Presentation

**Figure 1:**
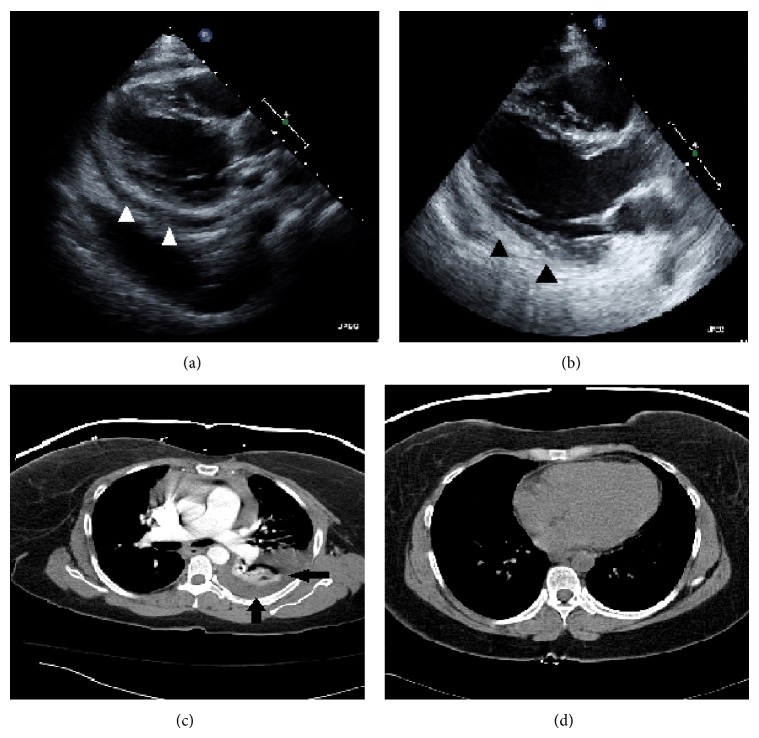
(a) Demonstrating pericardial effusion seen on echocardiogram, with (c) showing a large pleural effusion seen on computerized tomography. Both scans were obtained at second presentation in 2013. ((b) and (d)) Demonstrating complete resolution of both effusions at 3-month follow-up.

**Figure 2:**
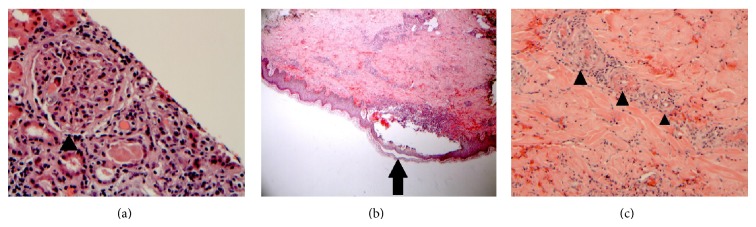
(a) Showing thickening of the glomerular capillary basement membrane (black arrow head). (b) Section of the skin demonstrates a neutrophil-rich subepidermal blister (black arrow) with (c) which shows leukocytoclastic vasculitis (black arrow heads) involving the blood vessels of the upper and deep dermis.

In January 2010, a 42-year-old African American female with medical history of hypertension and asthma presented with a rash of multiple red macules on the back of her thighs and legs, with occasional dark raised and painful spots. She also reported 5 months of diffuse asymmetrical arthritis involving the ankles, knees, elbow, hands, and shoulders. She reported chronic cocaine use, and her urinary drug screen (UDS) was positive for cocaine. On examination, the patient was afebrile. Skin showed diffuse palpable purpura, multiple 1 to 3 cm erythematous macules with areas of skin necrosis. Initial investigations showed creatinine of 1.8 mg/dL (normal 0.6–1.2 mg/dL) with nephrotic range proteinuria, ESR elevated at 96 mm/hr (normal 0–25 mm/hr), and normal complement levels. ANA, Anti-Smith and Anti-dsDNA were negative, but both c-ANCA and atypical p-ANCA were positive (titer 1 : 640 and 1 : 320, resp.). Biopsy of skin and kidney showed leukocytoclastic vasculitis and proliferative glomerulonephritis, respectively (Figure 2). SLE was suspected, and the patient was started on mycophenolate and prednisone, and the treatment continued for 36 months. The medications were stopped after her kidney function was back to normal. She had her prednisone doses tapered down slowly for extra 3 months beyond the treatment period. Patient was largely stable thereafter, except for persistently high p-ANCA. Patient was abstinent from cocaine between 2010 and 2013. She was free of symptoms; she denied any chest pain, shortness of breath, or skin rashes. Patient did not have any cocaine or other drugs like amphetamine or phencyclidine (PCP). In July 2013, she presented again with dyspnea, skin rash, and pleuritic chest pain that started abruptly two days ago. Pt was febrile, with multiple purpura. UDS was positive for cocaine and opiates. Coxsackie viral serology was negative. Chest X-ray and CT angiogram demonstrated moderate pericardial (Figure 1(a)) and pleural effusions (Figure 1(c)). Pleural fluid was exudative on thoracentesis and negative for tuberculosis and viral particles. ANA, Anti-Smith, and Anti-dsDNA were negative. Both proteinase-3 and p-ANCA were positive (titer 1 : 640). Patient was started on prednisone and discharged on a 6-month course. One month later, the patient reported symptom improvement, and the follow-up CT was negative for effusion (Figure 1(d)).

## 2. Discussion

Cocaine abuse affects more than 5 million people in the USA. Cocaine has been associated with multiple serological diseases, and vasculitis is a famous example. Several case reports have been discussing the recent rise of such cases and the potential role for cocaine adulterants in its pathogenesis. According to DEA, 69% of cocaine is being adulterated with levamisole [1], previously used as chemotherapeutic and immunomodulator for colon cancer and nephrotic syndrome [2]. Interestingly, levamisole has an immune stimulating effect that leads to production of both types of ANCA, as well as severe vascular damage up to skin necrosis [3]. Microscopically, levamisole can produce different patterns of vasculitis (leukocytoclastic and thrombotic) as seen in skin biopsies from children using levamisole for the treatment of nephrotic syndrome [4]. Levamisole can cause direct vascular damage through its antiangiogenic properties. It inhibits the proliferation, the differentiation, and capillary network formation of endothelial cells in vitro [5]. Levamisole derivatives disrupt endothelial network formation less efficiently than levamisole itself [6]. Levamisole has been reported to induce apoptosis in cultured endothelial cells [7]. Among cases describing cocaine-induced vasculitis, only 18 cases attributed to levamisole were reported, all within the past three years. None of these cases reported vasculitis associated with either pleural or pericardial effusion. On review of literature, only one case reported both pleural and pericardial effusion in a cocaine user without mention of concomitant vasculitis [8]. It described a large pleural and pericardial effusion in cocaine abuser. The proposed mechanisms were cocaine induced pulmonary embolism and cardiac toxicity from sympathetic system and renin-angiotensin system activation. Levamisole induced vasculitis was not included as a proposed mechanism. Our case is unique as it describes the development of both effusions in the setting of cocaine induced vasculitis with kidney and skin involvement. The pathological findings of skin biopsy done to our case are consistent with levamisole induced vasculitic pattern [4]. Between 2010 and 2013, the patient was abstinent from cocaine, and she was largely asymptomatic. The development of vasculitis and effusions was subsequent to a period of heavy cocaine intake. Interestingly, the patient reported using the same dealer in both settings, which can be the potential source of levamisole. We describe a syndrome of cocaine induced vasculitis with variable ANCA positivity, complicated with pleural and pericardial effusions (CIPP), possibly secondary to chronic levamisole adulteration. It shows a new aspect of cocaine complications that should be in consideration, especially in high-risk chronic abuse individuals who use street cocaine. As cocaine use increases, complications of levamisole will invariably rise. Prednisone is an option for treatment; however, no clear evidence supports its use.
